# Development of Improved DNA Collection and Extraction Methods for Handled Documents

**DOI:** 10.3390/genes14030761

**Published:** 2023-03-21

**Authors:** Ashley G. Morgan, Mechthild Prinz

**Affiliations:** 1 Department of Forensic Science, University of New Haven, 300 Boston Post Road, West Haven, CT 06516, USA; 2 Department of Sciences, John Jay College, 524 West 59th Street, New York, NY 10019, USA

**Keywords:** fingermarks, paper evidence, touch DNA, sample collection

## Abstract

Handwritten documents may contain probative DNA, but most crime laboratories do not process this evidence. DNA recovery should not impair other evidence processing such as latent prints or indented writing. In this study, single fingermarks on paper were sampled with flocked swabs, cutting, and dry vacuuming. In addition, two extraction methods were compared for the sample type. DNA yields were low across all methods; however, this work confirms the ability to recover DNA from paper and the usefulness of the vacuum sampling method combined with the Chelex-Tween method. Stability of touch DNA deposits were compared over an 11-month period to better understand degradation that may occur over time. No significant difference in DNA recovery was observed, suggesting DNA deposits on paper are stable over an 11-month span.

## 1. Introduction

Paper evidence may be recovered from a crime scene and is frequently encountered in forgery, aggravated harassment, and robbery investigations. Such evidence may be handwritten or typed, but likely contains DNA deposits due to handling.

Individuals vary in the amount of DNA they deposit on a surface [1]. Time since handwashing [1] and touching period [2] are all factors in the amount of DNA deposited; however, an individual’s shedder status may also play a role [1]. Which finger(s) or area of the hand contact the paper may impact the quantity of DNA recovered [3]. Regardless of which fingers touch the paper surface, brief contact with paper may transfer enough DNA to yield an interpretable DNA profile [2].

The variable composition of touch DNA deposits, which may include nucleated cells and cell-free DNAs from facial touching, sweat, and sebaceous secretions [4], can be attributed to activities prior to touch deposition. Larger quantities of DNA have been recovered from touched surfaces when tested individuals were allowed to perform activities after handwashing [5]. It has also been shown that more DNA accumulates with increasing time intervals after handwashing [6].

Optimization of sampling techniques is critical due to the variable nature of touch DNA deposition from fingermarks. Additionally, paper evidence may require analysis by multiple disciplines including DNA, questioned documents, and latent fingerprints. For this reason, evaluating a non-destructive sample collection method and extraction method may help in preserving evidence integrity and improving DNA recovery for this difficult material.

Few methods exist for non-destructive processing of paper evidence for trace DNA deposits from fingermarks. Traditional methods, including direct cutting [2] and wet/dry swabbing of paper [7], destroy the paper surface and prevent additional analyses. The porous and thin nature of paper requires imaginative sampling techniques to collect DNA from the paper surface, such as dry/dry swabbing [7] or vacuuming [8]. While the method selection is important, equally important is the swab itself, if that is the chosen collection method.

Previous work indicates that flocked swabs may improve DNA recovery from porous substrates when compared to traditional cotton swabs [9]. The highest extraction and recovery efficiencies have been observed for polyester Alpha^®^ swabs and nylon flocked swabs [10]. Fiber type and swab morphology may play a role in how DNA is collected from the surface and subsequently released in solution during purification.

Additionally, the analyst must address the PCR inhibitors present in the paper, if direct cutting, or on the paper surface, if swabbing. Use of an extraction kit tailored for plants may aid in lysis of cellular components and purification of DNA from plant-based samples, like paper [11]. Further purification may be necessary to combat bleaching agents and other components of paper that may inhibit PCR downstream.

While the method of DNA recovery is important, ensuring DNA degradation does not occur prior to sample collection is of equal importance. Evidence sampling may not occur within days or months following the original evidence collection and submission to the property and evidence unit.

This study compared several sampling methods, specifically, using flocked swabs to improve DNA recovery from handled documents, and tested a dry vacuum approach for single fingermarks. Traditional sampling by directly cutting from the paper was included as a benchmark. Furthermore, a stability study evaluated the stability of DNA fingermark evidence deposited on paper over 11 months.

## 2. Materials and Methods

Volunteers were recruited, and coded samples were collected in accordance with CUNY IRB#2017-0306 using an informed consent form.

Prior to sample collection from volunteers, half sheets of 8.5 in × 11 in 20 lb copy paper (Staples, Framingham, MA, USA) were irradiated with ultra-violet light for 15 min on each side and stored in a clean plastic container. An irradiated half sheet of copy paper was placed on a large lint-free wipe that had been placed on a clean laboratory table covered with new bench paper. Each volunteer was instructed to wash and dry their hands, touch their face and neck for 15 s, and rub their hands together for 15 s. This preparation was modeled after the Plaza et al. [12] “charging” protocol mimicking involuntary face touching. Once the volunteer had “charged” their hands, they were advised to press four fingers onto the half sheet of copy paper for five seconds. After deposition, the sheet was labeled with the individual’s code number and “L” or “R” with a pencil to designate handedness. The sample was then placed in a manila envelope labeled with the volunteer’s code number, date of collection, and sequence number. Only four fingers were targeted to eliminate donor hand size variability. While some volunteers would have been able to fit all five fingers on the paper half sheet, this did not work for others, which is why all volunteers were advised to press only four fingers on the paper during sample collection.

For the swab/direct cutting study, six pairs of samples were collected in sequence from three volunteers. For the vacuum study, again three volunteers were recruited, and three pairs of samples were collected from each individual. For the stability study, three pairs of samples from three volunteers were collected on two separate dates, for a total of six pairs of samples for each donor. Prior to the cutting, swabbing, or vacuuming recovery samples were stored at room temperature. After processing with one of the three methods, sample tubes were stored at room temperature until extraction.

### 2.1. Dry/Dry Flocked Swab Technique

A PurFlock Ultra polyester dry flocked swab (Puritan, Guilford, ME, USA) was rubbed on the entire surface of the half sheet of copy paper. The swab head was cut and placed into a 1.5 mL microcentrifuge tube (Promega, Madison, WI, USA). The swabbing procedure was repeated with a second polyester dry flocked swab. The swab head was added to the 1.5 mL microcentrifuge tube containing the first swab head.

### 2.2. Wet/Dry Flocked Swab Technique

A polyester flocked swab pre-moistened with 10 µL 0.1% Triton X-100 (Sigma-Aldrich, Allentown, PA, USA) was rubbed on the entire surface of the half sheet of copy paper. The swab head was cut into a 1.5 mL microcentrifuge tube. The swabbing procedure was repeated with a second dry polyester flocked swab and placed into the same 1.5 mL microcentrifuge tube containing the pre-moistened swab head.

### 2.3. Direct Cutting Technique

One-inch by one-inch cuttings were made from the half sheet of copy paper and placed into a 15 mL conical tube. Immediately prior to extraction, cutting samples were soaked with 800 µL of deionized water for 30 min at room temperature. The liquid portion was transferred to a QIAGEN Lyse and Spin (QIAGEN, Hilden, Germany) column-tube assembly and centrifuged for one minute at maximum speed (20,000× *g*). Once all liquid passed through the column, the column was discarded and tube containing the liquid was retained for extraction.

### 2.4. Vacuum Swab Technique

Using McLaughlin et al. [8] method as a guide, the vacuum swab technique was tested for wet or dry cotton swabs and glass or plastic pipettes.

**Figure 1:**
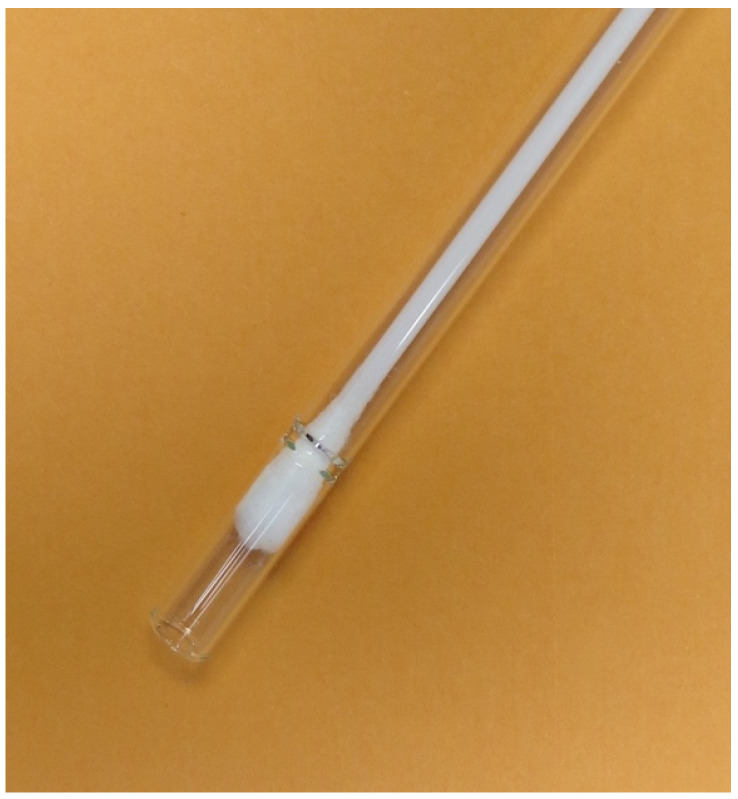
Swab inserted into glass Pasteur pipette. The tapered bottom of the apparatus was connected to a vacuum via plastic vacuum tubing. The cylindrical top containing the swab head was dragged across the paper surface to dry vacuum.

Prior to vacuum swabbing (Figure 1), the touched half sheet of copy paper was suspended on magnetic clips attached to a metal and wood base to prevent suctioning of the table surface while vacuuming the paper surface. The metal clips and metal sheet attached to the wood block were cleaned with DNA Away (ThermoFisher Scientific, Waltham, MA, USA), deionized water, and ethanol in between each sample. The laboratory table was cleaned and covered with bench paper.

#### 2.4.1. Glass Pasteur Pipette

A new clean Pasteur pipette was obtained, and the thin tip of the pipette was snapped off immediately prior to processing with the vacuum swab apparatus. The wood tip of a dry cotton swab (Puritan, Guilford, ME, USA) was snapped to shorten and inserted into the top of the Pasteur pipette. The cotton tip was situated just below the top. The tapered end of the altered pipette was attached to vacuum tubing connected to the vacuum of the M-Vac apparatus (M-Vac Systems, Inc., Sandy, UT, USA).

The vacuum was turned on and the pipette/swab apparatus was pulled across the sample sheet from left to right. This movement was repeated until the entire surface was vacuumed, then the vacuum was turned off. The cotton tip was cut into a microcentrifuge tube and stored at room temperature until DNA extraction. Samples from three volunteers were processed in this manner.

This procedure was repeated with additional samples from three volunteers using cotton swabs moistened with 10 µL of 0.1% Triton X-100 applied to the swab head.

#### 2.4.2. Plastic Pipette Tip

Prior to sample collection, 1000 µL plastic pipette tips were autoclaved. A cotton swab was shortened and inserted into the wide end of the pipette tip. The tapered end of the pipette tip was attached to vacuum tubing connected to a vacuum. Sample collection proceeded in the same manner as the glass Pasteur pipette. Samples from three volunteers were collected with dry swabs. An additional set of samples from three volunteers was collected using a swab moistened with 0.1% Triton X-100 as described in Section 2.4.1.

### 2.5. Extraction, Quantitation, Amplification

Half of the swabbing and direct cutting samples and all of the vacuum swab samples were extracted using a protocol based on the one described by Forsberg et al. [13]. To sample tubes, 200 µL of 5% Chelex 100 sodium form (Sigma-Aldrich, Allentown, PA, USA), 5 µL of 13.5 mg/mL Proteinase K (Promega, Madison, WI, USA) 2 µL of 10% Tween 20 (Sigma-Aldrich, Allentown, PA, USA), and 300 µL of deionized water were added. Additional deionized water was added to each tube to bring the volume up to 800 µL. Samples were incubated for 30 min at room temperature at the bench with vortexing or agitation occasionally. After 30 min, samples were incubated with a Multitherm shaker (Benchmark Scientific, Sayreville, NJ, USA) for 45 min at 56 °C shaking at 400 RPM. Next, samples were incubated at 98 °C with a Multitherm shaker set to no agitation for 10 min.

After incubation, each swab substrate was transferred to a spin basket (Promega, Madison, WI, USA) placed in a Dolphin tube, and centrifuged for five minutes at 1500× *g*. The collected liquid was transferred to the original sample tube. The substrate and spin basket-tube assembly was discarded. The DNA extract was concentrated using Microcon DNA Fastflow (MW100) filters (Millipore Sigma Burlington, MA, USA). Three hundred microliters of the liquid were transferred to the Microcon filter unit -tube assembly and centrifuged for 30 min at 500× *g*, the step was repeated for any remaining liquid. After the liquid on the Microcon filter was reduced to 5 µL of liquid, 30 µL of TE^−4^ buffer were added and the Microcon filter was inverted into a new collection tube and centrifuged for three minutes at 1000× *g*. The liquid collected in the tube was transferred to a new 1.5 mL tube and stored frozen until quantitation.

The remaining samples were extracted using the QIAamp DNA Investigator kit (QIAGEN, Hilden, Germany). Two and a half milliliters of TE^−4^ buffer were added to the direct cutting samples and incubated at room temperature for five minutes. The liquid was split into two microcentrifuge tubes and processed as described below.

Twenty microliters of 20 mg/mL Proteinase K (QIAGEN, Hilden, Germany) and 400 µL Buffer ATL were added to each sample and samples were vortexed briefly. Samples were incubated at 56 °C for one hour with shaking with a Multitherm shaker at 750 RPM. Following incubation, samples were briefly centrifuged. For samples containing swab heads, the material was transferred to a spin basket-tube assembly and centrifuged at 20,000× *g* for two minutes. The spin basket and material were discarded and 400 µL of Buffer AL was added to each tube. Samples were briefly vortexed and incubated for 10 min at 70 °C with shaking with a Multitherm shaker at 750 RPM. Samples were briefly centrifuged, then 200 µL of 96–100% ethanol was added to each sample. Samples were briefly vortexed and centrifuged. The entire liquid for each sample was transferred to a QIAamp MinElute column and centrifuged at 6000× *g* for one minute. The columns were transferred to new tubes. To each sample, 500 µL of Buffer AW1 was added. Samples were centrifuged at 6000× *g* for one minute and then columns were transferred to new collection tubes. Seven hundred microliters of Buffer AW2 was added to each sample and centrifuged for one minute at 6000× *g*. Columns were transferred to new collection tubes and 700 µL of ethanol was added. Samples were centrifuged at 6000× *g* for one minute. All columns were transferred to new collection tubes and centrifuged for three minutes at 20,000× *g* to dry column membranes. Columns were transferred to new collection tubes and the lids were opened. All samples were incubated at room temperature for 10 min at the bench. After incubation, 35 µL of TE^−4^ buffer was added to each column, tubes were recapped, and samples were incubated for five minutes at room temperature at the bench. Samples were centrifuged at 20,000× *g* for one minute and the liquid collected at the bottom of the tube was stored at −20 °C until quantitation.

DNA concentrations were determined by real-time PCR quantitation with the Quantifiler Trio kit on a 7500 Real-Time PCR instrument (Thermo Fisher Scientific, Waltham, MA, USA) according to the manufacturer’s specifications [14].

Swabbing, direct cutting, and stability study samples were amplified with the Globalfiler kit (Thermo Fisher Scientific, Waltham, MA, USA) targeting an input of 0.75 ng. Amplification reactions were prepared per the manufacturer’s specifications. The appropriate amounts of sample DNA and TE^−4^ buffer were added to each tube to achieve 0.75 ng of DNA in the maximum input volume of 15 µL. For samples with low quantities of DNA, 15 µL of sample DNA was added to each tube. Samples were amplified with 28 cycles using validated conditions [15].

Following amplification, samples were separated on the 3500 Genetic Analyzer instrument (Thermo Fisher Scientific, Waltham, MA, USA). DNA profiles were evaluated with Genemapper ID-X analysis software v1.6 (Thermo Fisher Scientific, Waltham, MA, USA) using a lower analytical threshold (LAT) of 30 relative fluorescence units (RFU), analytical threshold of 75 RFU, and a stochastic threshold of 350 RFU.

### 2.6. Stability Study

Samples were collected from three volunteers as described above using half sheets of copy paper irradiated with ultraviolet light for 15 min per side. Samples were stored at room temperature. Month 0 samples were extracted within a week of initial sample collection to set a baseline for each donor. A sample from each volunteer was swabbed and extracted once a month over 11 months. A half sheet from each volunteer was dry swabbed with a flocked swab and extracted using the Chelex-Tween method described above. Samples were quantified on the 7500 instrument using the Quantifiler Trio kit. Samples were amplified with Globalfiler and run on the 3500 Genetic Analyzer instrument as described above.

Statistical analyses were performed using R version 3.6.2 and R studio version 2022.12.0+353. See Morgan [16] for more details.

## 3. Results

### 3.1. Comparison of Sampling Technique

**Figure 2:**
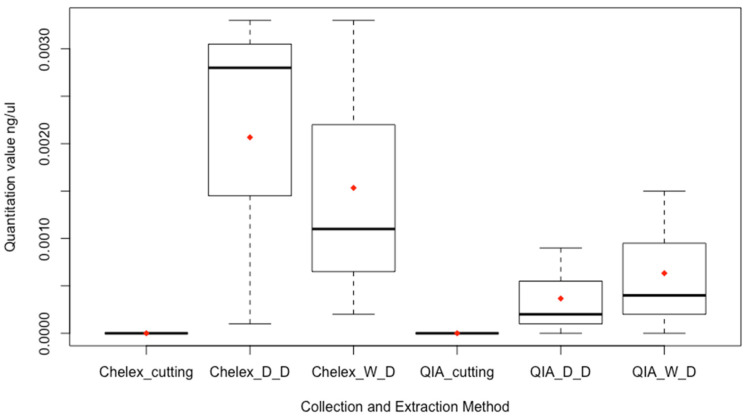
Box and whisker plot for the quantitation values for the collection and extraction methods, *n* = 3 each. The upper whisker indicates the maximum and the lower whisker indicates the minimum. The dark line indicates the median and the red diamond indicates the mean. Chelex indicates the Chelex-Tween extraction and QIA indicates the QIAamp DNA investigator kit extraction. Cutting indicates direct cutting sampling method. W_D indicates the wet/dry swabbing method and D_D indicates the dry/dry swabbing method. The dry/dry swabbing condition extracted with Chelex-Tween demonstrated the largest spread in quantitation values and the greatest mean.

No DNA was detected for the direct cutting sampling method for either extraction method (Figure 2). Higher mean quantitation values were observed for samples extracted with the Chelex-Tween method compared to the QIAamp method.

A multiple regression model evaluated the predictive value of extraction method and sampling method on quantitation value. No statistically significant effect (F(3,14) = 2.74, *p* = 0.082, R^2^ = 0.370) was observed for the model. None of the individual factors were statistically significant predictors for the quantitation value. (See Figure S1 for multiple regression summary).

All samples were amplified with the Globalfiler kit and DNA profiles were evaluated for number of peaks detected above the lower analytical threshold and analytical threshold.

**Figure 3:**
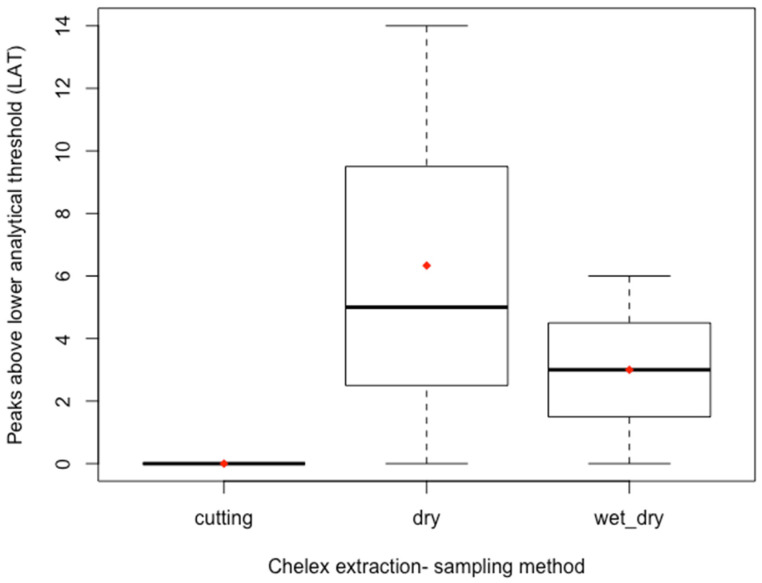
Box and whisker plot for the number of peaks observed above the lower analytical threshold (LAT) by sampling method, for Chelex-Tween extracted samples, *n* = 3 each. The red diamond indicates the mean. The upper whisker indicates the maximum and the lower whisker indicates the minimum. The dark line indicates the median. The dry swabbing condition demonstrated the greatest spread in the number of peaks observed above the LAT and a higher mean value than the other two sampling methods for the Chelex-Tween extraction method.

As expected based on the low DNA quantitation values, none of the samples yielded a full DNA profile. The number of peaks observed for all sampling conditions were compared for the Chelex-Tween extracted samples (Figure 3). The dry/dry swabbing condition showed the largest spread in number of peaks observed and the largest mean. Only a few peaks were observed in the wet/dry swabbing condition; the mean value was less than for the dry/dry condition. Direct cutting samples did not yield any peak information.

**Figure 4:**
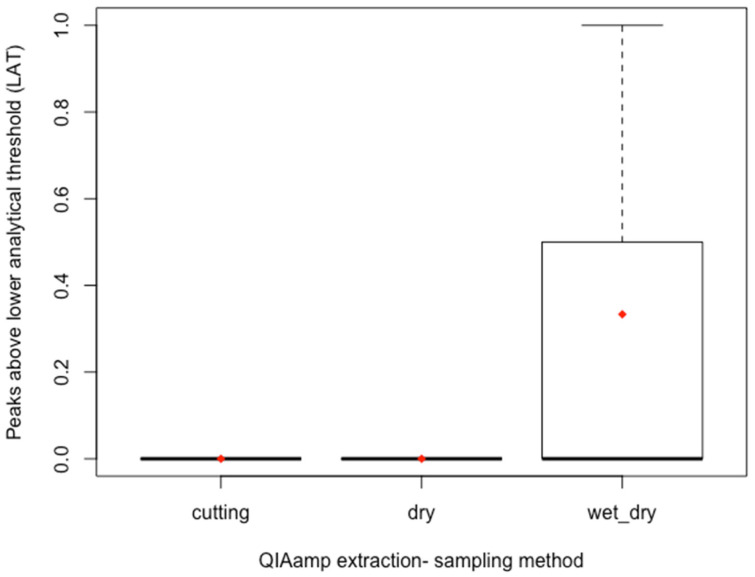
Box and whisker plot for the number of peaks observed above the lower analytical threshold (LAT) by sampling method, for QIAamp extracted samples, *n* = 3 each. The dark line indicates the median. The upper whisker indicates the maximum and the lower whisker indicates the minimum. The red diamond indicates the mean. The wet/dry swabbing condition demonstrated the largest spread in the number of peaks observed above the LAT and also the largest mean for QIAamp extracted samples.

Sampling methods for QIAamp extracted samples were also compared (Figure 4). Wet/dry swabbing performed the best of the QIAamp extracted samples. No peaks were observed above the lower analytical threshold for both the cutting and dry/dry swab conditions.

A statistically significant effect (F(4,13) = 12.19, *p* < 0.001, R^2^ = 0.789)) was observed for the multiple regression model evaluating the predictive value of sampling method, extraction method and quantitation value on the number of peaks observed above the lower analytical threshold. Quantitation value was a statistically significant predictor for the model (t = 5.38, *p* < 0.001). (See Figure S2 for multiple regression summary).

### 3.2. Vacuum Swab Technique

No statistically significant effect (F(2,19) = 2.01, *p* = 0.189, R^2^ = 0.309)) was observed in the multiple regression model evaluating the predictive value of pipette used for the vacuum swabbing and the swab being wet or dry on the quantitation value. (See Figure S3 for multiple regression summary).

Low DNA recovery was observed for a small subset of glass Pasteur pipette samples that had been extracted with ForensicGEM SexCrime kit (data not included). A spread in quantitation values was observed for all conditions evaluated (Table 1). Vacuum swab methods with the plastic pipette tip demonstrated the largest spread in quantitation values for both the dry and wet swab conditions. The glass pipette wet and dry swab conditions exhibited lower means and smaller spreads in DNA quantitation values.

Quantitation values were similar for both swab conditions using the glass pipette, with means that were close to zero. Evaluation by one-way analysis of variance (ANOVA) showed no statistically significant difference among the means (F(3) = 1.284, *p* = 0.344). (See Figure S4 for one-way ANOVA summary).

### 3.3. Stability Study

The stability of DNA from fingermarks deposited on paper was evaluated over an 11-month period. Samples collected from three donors were extracted, quantified, and amplified once a month for 11 months.

As seen in Table 2 below, the amount of DNA recovered remained consistent for each donor over 11 months. The quantitation value for month 2 for donor 002C demonstrated a quantitation value of 3.2568 ng/µL, which was much larger than all other values observed for this individual. A Grubbs test was performed to determine if there was a possible outlier in the data. The resultant G statistic was G = 5.82 and *p* < 0.001, which indicated the large value observed for month 2, donor 002C was an outlier. Once identified, the outlier was not included in further analysis. (See Figure S5 for Grubbs Test summary).

DNA averages varied by donor and exhibited large standard deviations, commonly seen in fingermark DNA studies. Donor 002C had larger DNA yields compared to donors 001C and 005C.

**Figure 5:**
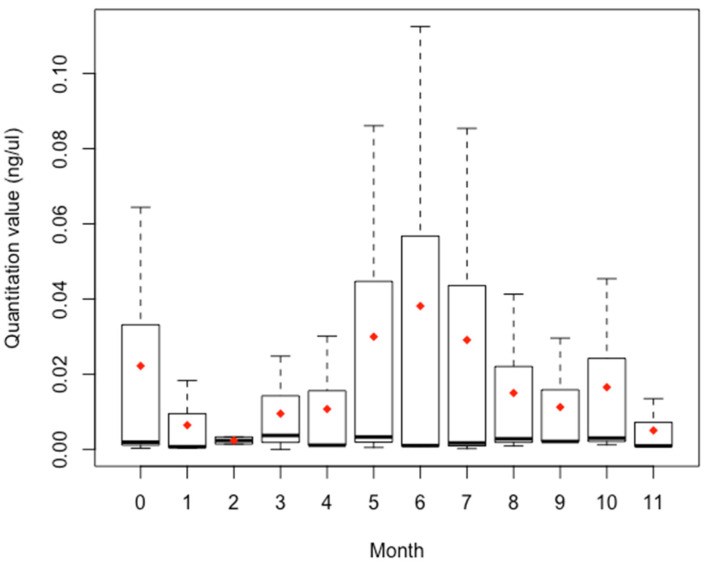
Box and whisker plot for quantitation values by month, *n* = 3, each. The dark line indicates the median and the red diamond indicates the mean. The upper whisker indicates the maximum and the lower whisker indicates the minimum. Month two showed the smallest range of quantitation values and lowest mean. The outlier for month two was not included in this figure.

A large spread in quantitation values among the three donors was observed for months five, six, and seven (Figure 5). Months two and ten showed the smallest spread in quantitation values. Median values across all months are consistent, while the means also show some variability month to month. A one-way analysis of variance (ANOVA) was performed to determine if there was a statistically significant difference among the means. No statistically significant difference was observed F(11) = 0.34 *p* = 0.97 among the monthly means. (See Figure S6 for one-way ANOVA summary).

**Figure 6:**
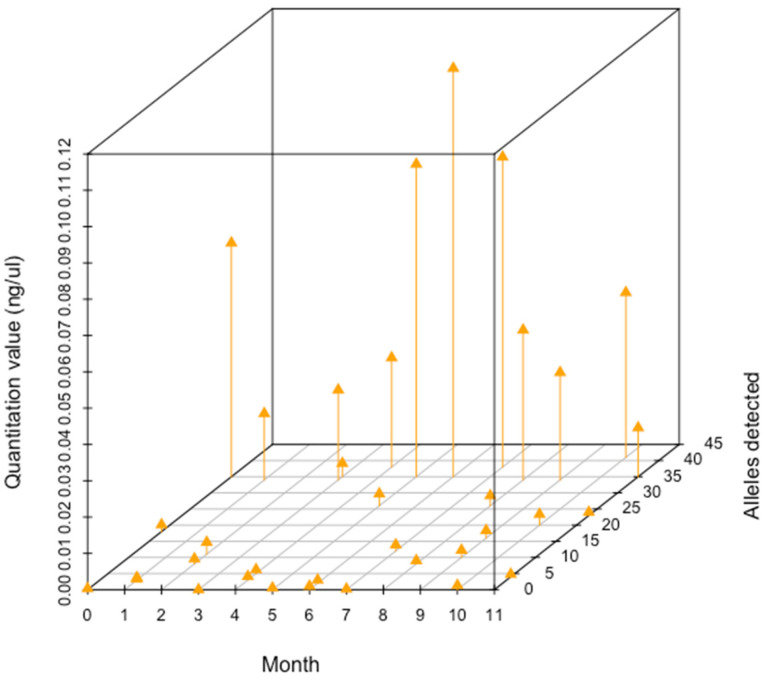
3-D scatterplot of quantitation values and alleles detected for each month, *n* = 3 per month. For each month, the quantitation value is on the left side of the figure and the number of alleles detected is on the right side of the figure.

While quantitation value may indicate success of a DNA collection and extraction method, profile quality and completeness are additional indicators of long-term stability of DNA from fingermarks on paper. The number of alleles detected, quantitation value, and the month were plotted in three dimensions to visualize the relationship among the factors (Figure 6). There was mixed success in the amount of DNA detected and the number of alleles detected for samples each month. In general, low quantity samples yielded a low number of alleles observed. As DNA quantity increased, the number of alleles observed varied.

A statistically significant effect (F(12,22) = 2.92, *p =* 0.014, R^2^ = 0.614) was observed for a multiple regression model evaluating the predictive value of the quantitation value and month of extraction on the number of alleles observed. The quantitation value was a statistically significant individual predictor for the model (t = 5.56, *p* < 0.001). Month of extraction was not a statistically significant predictor for the model. (See Figure S7 for multiple regression summary).

## 4. Discussion

Due to the limited sample size, handedness was not evaluated as a variable in this work. Additional consideration may be needed in a replicate study with a larger number of participants; however, no significant difference in DNA quantity was observed for right or left hands in work completed by Heftez et al. [17]. Other factors to be considered in additional studies are how to control finger “charging”, e.g., collecting the time since last face washing [5], and measuring the exact pressure of DNA deposition while placing fingers. Higher deposition pressure has been shown to transfer higher quantities of DNA to the surface of the substrate [17].

### 4.1. Direct Cutting and Swabbing Study

No statistically significant effect was observed for the extraction method and sampling method on the quantitation value obtained for the cutting/swabbing study. Similar results were observed by Parsons et al. [7], in which no significant difference in DNA quantity was observed for the wet/dry and dry/dry swabbing conditions. This result supports implementation of either sampling method; however, in a multi-discipline laboratory where other sections may analyze the evidence, the dry/dry sampling technique may be preferred.

Unlike Balogh et al. [2], no DNA profiles were obtained from paper cutting samples; however, the sample preparation method differed from this study and may partially explain the discrepancy. Sewell et al. [11] did observe low quantities of DNA from office paper cuttings which is consistent with the results observed in this study. However, Sewell et al. [11] observed a statistically significant increase in DNA recovery from paper cuttings extracted with the DNeasy plant mini kit compared to the QIAamp mini kit. Their result suggests the kit chemistry may improve sample purification and removal of PCR inhibitors from direct cuttings. In the future, comparison of the Chelex-Tween method with the DNeasy method may be worthwhile for the direct cutting sampling method; however, other commercially available extraction kits could improve DNA yields in swab samples from paper surfaces.

The quantitation value was a statistically significant predictor for the number of peaks observed above the lower analytical threshold. As expected, a higher quantitation value predicts an increased number of peaks observed in a DNA profile. A similar result was also observed in the stability study, the quantitation value was a statistically significant predictor for the number of alleles detected in the DNA profile.

### 4.2. Vacuum Swab Study

DNA recovery for both pipette materials was low and the multiple linear regression model exhibited no statistically significant effect for pipette material or swabbing method on the quantitation value when evaluated together. Similar to the direct cutting and swabbing study, none of the individual variables were statistically significant predictors. Overall, the vacuum swab samples had slightly higher DNA yields than the cutting and swabbing samples. This could mean the method is more efficient but may also be due to the difference in DNA donors for the samples. Additional comparison work with an identical pool of donors is recommended to confirm.

A large spread in quantitation values for the 1000 µL plastic pipette tip vacuum swab samples may be due to the experimenter observed difference in suction strength during sample collection. The plastic pipette tip had stronger suction while pulling across the sheet of copy paper and may have collected more DNA from the surface compared to the glass pipette. A disadvantage of the plastic pipette tip is that the stronger suction made it harder to manipulate the swab-pipette tip apparatus while vacuuming the paper surface. To combat the strong suction, it may be worth testing the vacuum collection method with a filtered pipette tip and removing the filter for lysis and purification as recently described for fabric [18]. Use of the filter rather than a swab head may decrease the strength of suction as it is dragged across the document.

The vacuum DNA recovery here was also low compared to the work by McLaughlin et al. [8], but both studies demonstrated the utility of the vacuum swab technique. The lower yield can be explained by a difference in study design with only half sheets of paper and four individual prints here and a full letter size sheet with handwritten text in the other study. A study on mock casework handwritten documents showed a 67% success rate of interpretable STR profiles in copy paper and a 92% success rate for notepad paper [16]. Further modifications to the method should be explored. Bruijns et al. [10] noted a maximum extraction efficiency with nylon flocked swabs at approximately 48%, while the cotton swab performance was approximately 21%. Substitution of the cotton swab for a nylon flocked swab for the vacuum method may improve DNA recovery in subsequent studies. An alternative may be using inverted filter tips, but not all filter tips may show the same DNA recovery [18].

### 4.3. Stability Study

One of the three donors for the stability study demonstrated consistently large quantities of DNA from the paper samples, which is consistent with the literature regarding shedder status and variability in DNA deposition [1]. While differences in donors were observed, there was no statistically significant difference among the monthly means, which suggests that DNA from fingermarks remains stable through 11 months of storage at room temperature. This result is in line with previous work by Wurmbach and Ostojic [19] evaluating the effect of storage time on STR typing success. No significant difference in STR success was observed.

## 5. Conclusions

This work demonstrates the potential for paper evidence processing using the dry vacuum technique [8] in conjunction with the Chelex-Tween extraction method [13]. The materials for both the dry vacuum technique and Chelex-Tween extraction are inexpensive and the methods are simple to perform. For crime laboratories that do not currently process paper evidence, implementation of this technique requires minimal low-cost equipment and materials for internal validation and training of personnel. From a workflow point of view the dry vacuum method has the advantage that it preserves other evidence types such as indented writing and fingermarks [8]. As this study has shown, vacuuming is more effective than other DNA recovery methods, which means it can be used to collect DNA prior to any other examinations thus avoiding DNA loss and DNA contamination.

The authors acknowledge the small sample sizes for each study and suggest additional work with a larger sample size to confirm findings presented in this paper. Replicate studies for the sampling method comparison and the stability study should include improved sample preparation, a larger pool of volunteers, standardized pressure during fingermark deposition, and use of “realistic” mock evidence samples to provide additional insight.

## Figures and Tables

**Table 1:** Swabbing method and quantitation value (ng/µL).

	Glass		Plastic	
	Vac Dry	Vac Wet	Vac Dry	Vac Wet
Chelex-Tween	0.0002	0.0001	0.0001	0.0002
	0.0004	0.0006	0.0021	0.003
	0.0002	0.0016	0.0062	0.0039

**Table 2:** Quantitation value by donor by month. The value for donor 002C for month 2 was an outlier and was not included in the average and standard deviation calculations.

Month	Donor 001C (ng/µL)	Donor 002C (ng/µL)	Donor 005C (ng/µL)
0	0.0019	0.0644	0.0003
1	0.0007	0.0183	0.0003
2	0.0033	3.2568	0.0014
3	0.0037	0.0248	0
4	0.0011	0.0301	0.001
5	0.0033	0.0861	0.0005
6	0.001	0.1125	0.0009
7	0.0002	0.0854	0.0017
8	0.0009	0.0413	0.0028
9	0.0021	0.0296	0.002
10	0.0012	0.0454	0.003
11	0.0009	0.0135	0.0007
Average	0.0016	0.0501	0.0012
Standard Deviation	0.0011	0.0324	0.0009

## Data Availability

The data presented in this study are available upon request from the corresponding author.

## Supplementary Materials

The following supporting information can be downloaded at: https://www.mdpi.com/article/10.3390/genes14030761/s1, Figure S1: Summary of multiple regression for extraction method and sampling method predictors for quantitation value; Figure S2: Summary of multiple regression for extraction method, sampling method, and quantitation value predictors for total number of peaks observed above the lower analytical threshold; Figure S3: Summary of multiple linear regression for pipette material and wet and dry swabbing as predictors for quantitation value for dry vacuum method; Figure S4: One-way ANOVA summary for vacuum collection method comparison; Figure S5: Grubbs Test summary for stability study; Figure S6: One-way ANOVA summary for stability study; Figure S7: Multiple regression summary for stability study.
