# Supplementary material for: Development of Improved DNA Collection and Extraction Methods for Handled Documents
Source: Genes (Basel). 2023 Mar 21;14(3):761. doi: 10.3390/genes14030761 (PMC10048427; doi:10.3390/genes14030761)
Supplement: Supplementary file 1 [file genes-14-00761-s001.zip › Supplementary Figures.pdf]

Supplemental Figures

```
Call:
lm(formula = Quant ~ Emethod + Sampling)

Residuals:
       Min         1Q     Median         3Q        Max 
-0.0015500 -0.0005458 -0.0003333  0.0004333  0.0017833 

Coefficients:
                   Estimate Std. Error t value Pr(>|t|)  
(Intercept)       0.0004333  0.0004853   0.893   0.3870  
EmethodQIAamp    -0.0008667  0.0004853  -1.786   0.0958 .
Samplingdry       0.0012167  0.0005944   2.047   0.0599 .
Samplingwet_dry   0.0010833  0.0005944   1.823   0.0898 .
---
Signif. codes:  0 ‘***’ 0.001 ‘**’ 0.01 ‘*’ 0.05 ‘.’ 0.1 ‘ ’ 1

Residual standard error: 0.001029 on 14 degrees of freedom
Multiple R-squared:  0.3703,	Adjusted R-squared:  0.2353 
F-statistic: 2.744 on 3 and 14 DF,  p-value: 0.08246
```

Figure S1. Summary of multiple regression for extraction method and sampling method predictors for quantitation value

For EmethodQIAamp: ChelexTween=0, QIAamp=1
For Samplingdry: cutting=0, dry=1, wet_dry=0
For Samplingwet_dry: cutting=0, dry=0, wet_dry=1

```
Call:
lm(formula = AboveLAT ~ Quant + Sampling + Emethod)

Residuals:
    Min      1Q  Median      3Q     Max 
-2.7012 -0.5216  0.0901  0.3894  4.9794 

Coefficients:
                  Estimate Std. Error t value Pr(>|t|)    
(Intercept)        0.35655    0.91521   0.390 0.703155    
Quant           2638.73287  490.26756   5.382 0.000125 ***
Samplingdry       -0.04379    1.24279  -0.035 0.972426    
Samplingwet_dry   -1.19196    1.21277  -0.983 0.343625    
EmethodQIAamp     -0.71310    0.98642  -0.723 0.482536    
---
Signif. codes:  0 ‘***’ 0.001 ‘**’ 0.01 ‘*’ 0.05 ‘.’ 0.1 ‘ ’ 1

Residual standard error: 1.888 on 13 degrees of freedom
Multiple R-squared:  0.7895,	Adjusted R-squared:  0.7248 
F-statistic: 12.19 on 4 and 13 DF,  p-value: 0.0002445
```

Figure S2. Summary of multiple regression for extraction method, sampling method, and quantitation value predictors for total number of peaks observed above the lower analytical threshold.

For EmethodQIAamp: ChelexTween=0, QIAamp=1
For Samplingdry: cutting=0, dry=1, wet_dry=0
For Samplingwet_dry: cutting=0, dry=0, wet_dry=1

```
Call:
lm(formula = Quant ~ as.factor(Glass_plastic) + as.factor(W_D))

Residuals:
       Min         1Q     Median         3Q        Max 
-0.0024667 -0.0004417 -0.0002000  0.0005667  0.0036333 

Coefficients:
                           Estimate Std. Error t value Pr(>|t|)  
(Intercept)               5.000e-04  8.918e-04   0.561   0.5887  
as.factor(Glass_plastic)1 2.067e-03  1.030e-03   2.007   0.0757 .
as.factor(W_D)1           3.333e-05  1.030e-03   0.032   0.9749  
---
Signif. codes:  0 '***' 0.001 '**' 0.01 '*' 0.05 '.' 0.1 ' ' 1

Residual standard error: 0.001784 on 9 degrees of freedom
Multiple R-squared:  0.3092,	Adjusted R-squared:  0.1557 
F-statistic: 2.014 on 2 and 9 DF,  p-value: 0.1893
```

Figure S3 Summary of multiple linear regression for pipette material and wet and dry swabbing as predictors for quantitation value for dry vacuum method

For Glass_plastic: glass=0, plastic=1
For W_D: dry=0, wet=1

```
> vac_aov2<-aov(Quant~Collection.method)
> summary(vac_aov2)
                  Df    Sum Sq   Mean Sq F value Pr(>F)
Collection.method  3 1.347e-05 4.490e-06   1.284  0.344
Residuals          8 2.798e-05 3.498e-06
```

Figure S4 One-way ANOVA summary for vacuum collection method comparison

```
	Grubbs test for one outlier

data:  Quant
G = 5.82522, U = 0.00278, p-value <2e-16
alternative hypothesis: highest value 3.2568 is an outlier
```

Figure S5 Grubbs Test summary for stability study

```
                   Df  Sum Sq Mean Sq F value Pr(>F)
as.factor(month) 11 0.00396 0.00036    0.34   0.97
Residuals        23 0.02449 0.00106
```

Figure S6 One-way ANOVA summary for stability study

```
Call:
lm(formula = alleles.detected ~ Quant + as.factor(month))

Residuals:
    Min      1Q  Median      3Q     Max
-19.115  -7.907   0.077   6.514  16.571

Coefficients:
                   Estimate Std. Error t value Pr(>|t|)
(Intercept)          8.5886     6.8411    1.26     0.22
Quant              408.9198    73.5267    5.56  1.4e-05 ***
as.factor(month)1    2.1140     9.4666    0.22     0.83
as.factor(month)2   -0.0496    10.6053    0.00     1.00
as.factor(month)3   10.5266     9.4417    1.11     0.28
as.factor(month)4    2.3556     9.4331    0.25     0.81
as.factor(month)5   -0.5093     9.4127   -0.05     0.96
as.factor(month)6  -11.8488     9.4681   -1.25     0.22
as.factor(month)7   -3.8215     9.4091   -0.41     0.69
as.factor(month)8    7.9442     9.4103    0.84     0.41
as.factor(month)9    6.8178     9.4299    0.72     0.48
as.factor(month)10   4.9839     9.4046    0.53     0.60
as.factor(month)11  10.0198     9.4798    1.06     0.30
---
Signif. codes:  0 ‘***’ 0.001 ‘**’ 0.01 ‘*’ 0.05 ‘.’ 0.1 ‘ ’ 1

Residual standard error: 11.5 on 22 degrees of freedom
Multiple R-squared:  0.614,     Adjusted R-squared:  0.404
F-statistic: 2.92 on 12 and 22 DF,  p-value: 0.0142
```

Figure S7 Multiple regression summary for stability study
